# Multiplex ddPCR: A Promising Diagnostic Assay for Early Detection and Drug Monitoring in Bovine Theileriosis

**DOI:** 10.3390/pathogens12020296

**Published:** 2023-02-10

**Authors:** Shweta Murthy, Akash Suresh, Debabrata Dandasena, Sakshi Singh, Madhusmita Subudhi, Vasundhra Bhandari, Vandna Bhanot, Jaspreet Singh Arora, Paresh Sharma

**Affiliations:** 1 National Institute of Animal Biotechnology, Hyderabad 500032, India; 2 Graduate Studies, Regional Centre for Biotechnology (RCB), Faridabad 121001, India; 3 National Institute of Pharmaceutical Education and Research (NIPER), Hyderabad 500037, India; 4 Disease Investigation Laboratory, LUVAS (Hisar), Haryana 125011, India; 5 School of Animal Biotechnology, GADVASU, Chandigarh 141012, India

**Keywords:** bovine theileriosis, *Theileria annulata*, ddPCR, diagnostic, drug resistance

## Abstract

Accurate quantification based on nucleic acid amplification is necessary to avoid the spread of pathogens, making early diagnosis essential. Droplet digital PCR (ddPCR) stands out for absolute parasite quantification because it combines microfluidics with the TaqMan test. This helps deliver maximum accuracy without needing a reference curve. This study assessed the efficacy of ddPCR as a detection tool for the bovine theileriosis (BT) caused by *Theileria* parasites. We developed and validated a duplex ddPCR method that detects and quantifies the *Theileria* genus (18S rRNA) and identifies clinically significant *Theileria annulata* parasites (TaSP) in experimental and clinical samples. ddPCR was shown to be as effective as qPCR throughout a 10-fold sample dilution range. However, ddPCR was more sensitive than qPCR at lower parasite DNA concentrations and reliably assessed up to 8.5 copies/µL of the TaSP gene in the infected DNA (0.01 ng) samples. The ddPCR was very accurate and reproducible, and it could follow therapeutic success in clinical cases of theileriosis. In conclusion, our ddPCR assays were highly sensitive and precise, providing a valuable resource for the study of absolute parasite quantification, drug treatment monitoring, epidemiological research, large-scale screening, and the identification of asymptomatic parasite reservoirs in the pursuit of BT eradication.

## 1. Introduction

Human and animal parasite diseases spread by vectors are potentially fatal and cause massive economic losses if not promptly treated. Bovine theileriosis (BT) is a vector-borne disease linked to fatalities in tropical and subtropical countries [1]. Even though several *Theileria* parasite species have been identified in the field, just two pathogenic species, *Theileria annulata* and *Theileria parva*, are responsible for most economic losses in the cattle industry [2,3]. In India, *T. annulata* is the primary cause of BT, costing the dairy industry an estimated loss of $1.29 billion per annum [4]. Resistance to buparvaquone (BPQ) in the field has impeded the management of this parasite, the treatment of which is entirely reliant on it [5,6,7,8]. It has been shown that early parasite detection is important for the BPQ to efficiently eliminate the *Theileria* parasite [5]. In cattle, delayed diagnosis might result in deadly lymphoproliferative disease. Because early parasite identification is critical for parasite management, precise and accurate quantification of *T. annulata* parasites is essential for mass screening and treatment protocols to be efficiently applied in the field.

The current gold standard for diagnosing *T. annulata* parasites is based on disease symptoms paired with light microscopy (LM) [9]. Low blood parasite numbers make early microscopy identification difficult, and the presence of identical parasites, such as *Babesia*, may lead to false conclusions [10,11]. Several molecular diagnostic techniques based on *T. annulata*-specific genes have been developed to identify *Theileria* parasites, enabling the disease to be diagnosed at low parasite counts and with greater sensitivity than the LM [11,12,13,14,15,16]. Our group had recently developed a real-time PCR method for determining the parasite-to-host ratio and parasitemia in clinical samples of BT [17]. Despite its superior sensitivity and accuracy compared to previous molecular or serological approaches used in the field, there are still areas where the quantitative test may be improved. One key disadvantage of existing qPCR techniques is their reliance on the reference standard curve, which makes comparing qPCR findings between labs challenging [17,18,19,20,21,22]. Developing an assay with higher performance at low doses without the requirement for a reference standard curve should increase the detection of infection, hence facilitating case identification and disease monitoring. ddPCR is a breakthrough DNA quantification technique that avoids dependence on the reference standard curve and is effective in diagnosing and quantifying various human and animal pathogens, including vector-borne infections [23,24,25,26,27,28,29,30,31,32,33,34,35]. With more consistent findings across technical replicates and higher sensitivity than qPCR, ddPCR has been gaining traction as a viable pathogen identification and quantification option.

We evaluated the sensitivity of ddPCR to identify and quantify *T. annulata* parasites in clinical and in vitro samples. The analytical and diagnostic sensitivity and specificity of ddPCR were compared to the qPCR iteration targeting the microschizont stage-specific parasite gene TaSP to evaluate the technique’s efficacy. In addition, we devised a multiplex test that can detect *T. annulata* parasites and other *Theileria* species in the field.

## 2. Materials and Methods

### 2.1. Sample Collection, DNA Isolation, and Theileria-Specific PCR

Bovine blood samples (N = 151) were collected from Haryana, India, which were suspected for BT based on symptoms and LM (Geimsa Staining). Approximately 2 mL of blood was collected into EDTA-coated vacutainer tubes (BD) with the assistance of veterinarians. The standard phenol-chloroform-isoamyl alcohol procedure was utilized to isolate DNA from blood. The DNA concentration and integrity were confirmed through nanodrop and agarose gel electrophoresis. The *T. annulata* infection was confirmed using polymerase chain reaction (PCR) by amplifying *T. annulata* specific primers for the TaSP gene. We used gDNA from an in vitro cultured strain of *Theileria*-infected bovine cells (TA) as a positive control from our lab.

The PCR conditions for the primers using NEB Taq Polymerase (M0273) mentioned in Table 1 were as follows:

TaSP: 95 °C for 3 min, followed by 35 cycles of 95 °C for 30 s, 60 °C for 30 s, and 72 °C for 30 s, and a final extension of 3 min at 72 °C.

### 2.2. In Vitro Culture of the T. annulata Infected Bovine Lymphocyte Cell Line (TA)

*T. annulata*-infected lymphocytes were cultured in RPMI 1640 medium with 10% FBS and Pen/Strep (100 µg/mL) solution at 37 °C and 5% CO_2_, as described earlier in Dandasena et al., 2018. The standard phenol-chloroform-isoamyl alcohol procedure was utilized to isolate DNA from the cell line. The DNA concentration and integrity were confirmed using nanodrop and agarose gel electrophoresis.

### 2.3. ddPCR

To detect *Theileria* infection, the ddPCR approach was optimized first in TA cells and then tested in field samples (N = 151). The primers and probes for this study were designed and obtained from Eurogentec, Belgium and their specificity was confirmed using NCBI BLAST.

The total volume of the ddPCR mixture was kept at 20 µL which included primers (0.9 µM forward and reverse primers), probes (0.2 µM), DNA (100 ng), and 1× ddPCR super mix (Bio-Rad, Hemel Hempstead, UK). The reaction was carried out in duplicate. Droplet creation, reading, and data analysis for ddPCR were performed using the Bio-Rad QX100, as previously reported [29].

### 2.4. qPCR

The TaSP gene was used in a qPCR test to assess the analytical and diagnostic performance of the ddPCR results [17]. The qPCR experiment was conducted on TA cell line DNA using a previously described technique [17]. Table 1 lists the primers that were utilized for the qPCR.

### 2.5. TaSP Quantification through qPCR

The amplified product of TaSP was cloned into a pBSK plasmid using the TA cloning method as described earlier (https://www.thermofisher.com/in/en/home/life-science/cloning/ta-cloning-kits.html (accessed on 22 March 2022)). Later, pBSK was transformed into the *E. coli* cells (Top10), and cloned plasmids were screened for the presence of TaSP genes. Following that, these cloned pBSK-TaSP plasmids were serially diluted and qPCR quantified for Cq values using SYBR green at corresponding copies/µL. Based on these values, a standard graph was generated from which the copies/µL of test samples were derived.

### 2.6. Monitoring Treatment Response

To assess the effectiveness of the anti-*Theilerial* therapy, we examined the usefulness of the ddPCR in monitoring the treatment. In a 6-well plate, TA cells were seeded at a density of 2 × 10^5^ cells with and without treatment with BPQ for 72 h at a concentration of 200 ng/mL. DNA was extracted from treated and untreated samples and tested using ddPCR with the help of TaSP gene primers and probes. The experiment was carried out in triplicate.

### 2.7. Ethical Approval and Informed Consent

We initially acquired verbal permission from the owners before collecting blood from animals. In India, however, acquiring blood samples of less than 5 mL is not prohibited and does not need the consent of an ethical committee. Qualified veterinarians collected blood samples.

### 2.8. Statistical Analysis

GraphPad Prism 7.0 and Microsoft Excel 2019 were used for linear regression analysis of real-time PCR standard curves. Using QuantaSoftTM analysis software 1.7.4.0917 and a fluorescence amplitude threshold, positive and negative droplets were distinguished (Bio-Rad). Using the autofluorescence amplitude threshold mode, all observations were obtained. The retrieved ddPCR data were then analyzed in Microsoft Excel to provide means and standard deviations.

## 3. Results

### 3.1. Comparing the Sensitivity and Accuracy of ddPCR Assay for T. annulata Detection

**Figure 1:**
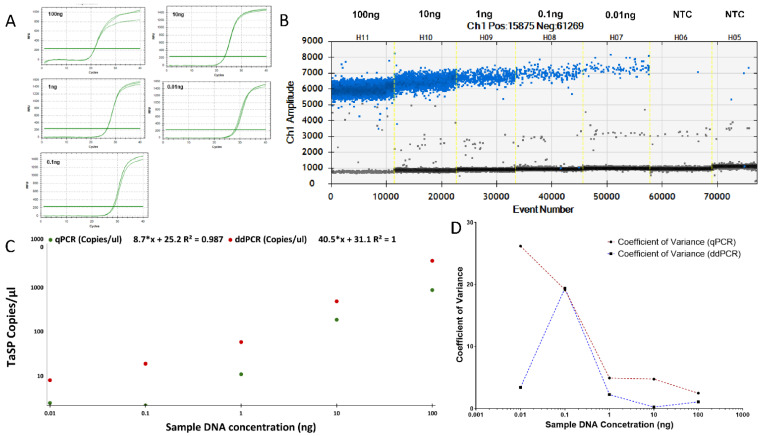
Quantification of TaSP gene using ddPCR and qPCR: The output generated by the (**A**) qPCR and (**B**) ddPCR instruments upon the usage of sample DNA at varying concentrations ranging from 0.01 ng to 100 ng, where the ddPCR output is based on the number of positive droplets denoted by blue dots, where each dot represents a droplet. (**C**) TaSP copies/µL was checked in *Theileria* infected sample’s DNA at varying concentrations ranging from 100 ng to 0.01 ng through 10-fold dilutions (*p*-value = 0.002). (**D**) Coefficient of variance (CV) comparison between ddPCR and qPCR values for copies/µL of TaSP gene.

We compared the sensitivity of ddPCR and qPCR for identifying *T. annulata* parasites by utilizing TaSP gene primers. TA cell DNA was serially diluted in water to assess the sensitivity of TaSP gene primers ranging from 100 ng to 0.01 ng (Figure 1A,B). Compared to qPCR (regression slope y = 8.7*x + 25.2 R^2^ = 0.987), ddPCR quantification precisely paralleled the dilution stages (regression slope y = 40.5*x + 31.1 R^2^ = 1) (Figure 1C). At 0.01 ng of DNA, qPCR found 2.5 TaSP gene copies/µL. However, 8.2 copies/µL were detected using ddPCR in the same sample. As shown by the correlation coefficient, the sensitivity of the ddPCR reaction is greater than that of the qPCR when the number of parasite DNA copies is low, as measured by the number of TaSP copies (R^2^). The repeatability of the ddPCR and qPCR assays was assessed by comparing their coefficient of variation (CV) over a range of parasite concentrations using TaSP gene quantification. Comparing the CV of ddPCR (3.4%) to that of qPCR (26.1%) readings of the same samples at lower concentrations (0.01 ng) demonstrates that ddPCR findings are more consistent and reproducible (Figure 1D). When the number of parasite DNA copies is low, linear regression analysis and CV demonstrate that ddPCR is preferable to qPCR.

### 3.2. Multiplex ddPCR Assay for Diagnosing Theileria Species and T. annulata Parasites from the Field

**Figure 2:**
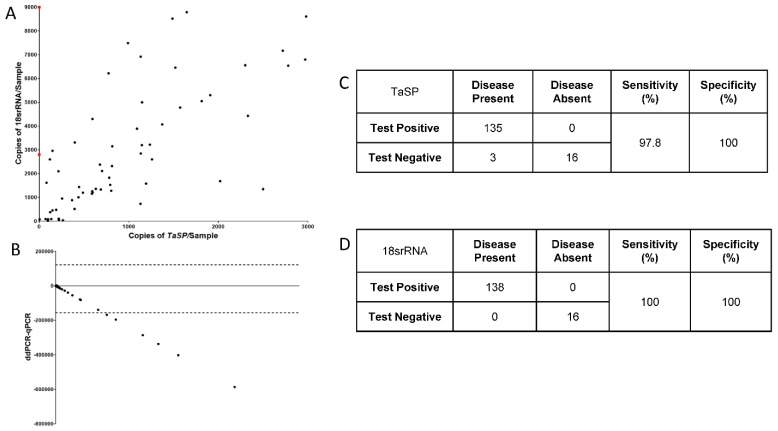
Screening of *Theileria* parasite in clinical bovine samples: (**A**) The scatter plot represents the copies/µL of TaSP and 18s rRNA in clinical samples (N = 151). (**B**) Bland-Altman plot comparing the efficiency of ddPCR-based diagnosis in comparison to qPCR across the samples (N = 151). The efficiency of both primers. (**C**,**D**) Sensitivity and specificity of TaSP and 18s rRNA primers when used in ddPCR for screening clinical samples.

We established a duplex ddPCR test using TaSP and 18s rRNA to diagnose *Theileria* species and *T. annulata* parasites in field samples. Using 18s rRNA primers, the multiplex test can help identify all *Theileria* genera, whereas TaSP confirms *T. annulata* infections. The ddPCR assay for the two genes was optimized using TA cell DNA. To determine the diagnostic performance of the 18s rRNA and TaSP gene primers, 151 DNA samples from potentially infected animals were collected from the field (Figure 2A). In the ddPCR experiment using duplex primers, the optimal annealing temperature of 60 °C enabled a distinct separation between DNA-positive and DNA-negative droplets. The 18S rRNA gene was identified in 138 of these samples, whereas the TaSP gene was identified in 135. In 13 samples, earlier identified as positive for theileriosis based on symptoms or LM, no amplification was seen with any primer. In the ddPCR samples, the 18S rRNA and TaSP primers sensitivity was 100% and 97.8%, respectively (Figure 2C,D). Across all tests, 100% of all clinical samples were concordant. Both the positive and negative samples were accurately identified. Using the TaSP template, a Bland-Altman plot was used to compare the quantitative differences between ddPCR and qPCR (Figure 2B). When the number of copies of parasite DNA was minimal, there were no quantitative differences between ddPCR and qPCR. Nonetheless, quantitative differences between the two techniques became more evident when the number of parasites increased.

### 3.3. Treatment Efficiency of the ddPCR Assay

**Figure 3:**
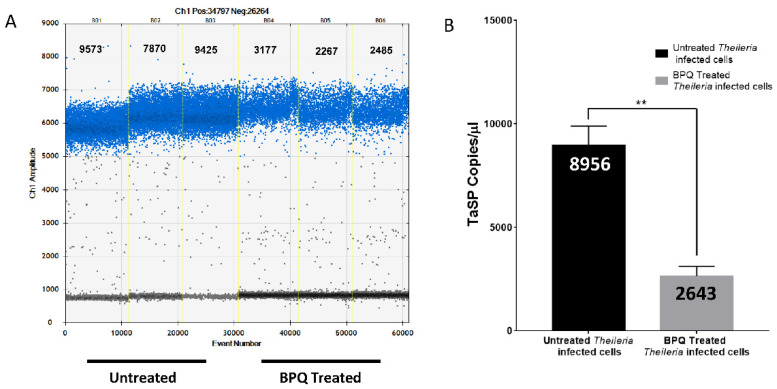
Detection of parasite DNA reduction upon BPQ treatment using ddPCR: (**A**) B01, B02, and B03 are the Ch1 amplitude of untreated samples while the B04, B05, and B06 are the same as BPQ treated samples. (**B**) Graph shows TaSP copies/µL in BPQ treated vs. untreated TA cells. Paired *t*-Test ** *p* < 0.01 (*p*-value = 0.0038).

Identifying *T. annulata* infection in the field is challenging, making tracking the treatment response even more difficult. Since some of the treated animals carrying the parasites remain undetected, they become carriers for ticks, leading to further disease propagation. ddPCR was performed on DNA samples from treated and untreated *Theileria*-infected cell lines (TA) with BPQ to evaluate the treatment’s effectiveness. After 72 h of BPQ therapy, DNA was extracted from the cell lines to monitor the parasite growth based on the TaSP gene expression. Using ddPCR, the number of TaSP copies/µL before and after BPQ treatment was determined. The number of parasite DNA copies dramatically reduced in treated samples in contrast to untreated ones. After 72 h, TaSP gene copies in DNA were measured to be only 2643 copies/µL in treated samples in comparison to 8956 copies/µL in control cell lines. (Figure 3A,B).

## 4. Discussion

Domestic and wild animals carry several *Theileria* species in tropical and subtropical regions; however, *T. annulata* and *T. parva* pose the most significant concern due to their ability to cause severe sickness and high mortality in cattle. *T. annulata* is the most concerning *Theileria* species in Indian cattle and requires prompt diagnosis and treatment. Molecular techniques based on the PCR have been developed to detect and quantify *Theileria* species and the *T. annulata* parasite [11,12,15,16,17,18,19,20,21,22,36,37,38]. Although qPCR-based techniques have shown promise in detecting *Theileria* infections, absolute quantification of parasites remains challenging due to qPCR’s dependence on reference standard curves. Furthermore, because different laboratories use different reference standard curves, comparing qPCR results from varying locations is difficult. A novel, sensitive, absolute quantitative test for identifying and quantifying *Theileria* parasites at the genus and species levels is required for detecting early infection and asymptomatic carrier animals. In this study, multiplex droplet digital PCR was employed to offer a reference standard curve-free approach for detecting and quantifying *Theileria* parasites at the genus and *T. annulata* at the species levels. Multiplex PCR using 18s rRNA identified the *Theileria* genus, and TaSP suggests *T. annulata* infection. The decision to treat or not treat an animal based on a TaSP diagnosis will aid in the fight against antibiotic resistance. It is because, unlike *T. annulata*, other *Theileria* parasites are not life-threatening and may be eliminated without medical intervention.

To the best of our knowledge, this is the first study to use ddPCR to quantify livestock-related *Theileria* parasites. Our data show that ddPCR is sensitive and accurate in identifying *T. annulata* parasites. When comparing ddPCR with qPCR, the former proved to be more reliable and sensitive while measuring parasite DNA at low sample concentrations. At lower parasite DNA, ddPCR exhibited considerably less variance than qPCR between technical replicates. Because ddPCR does not need a standard curve, findings from different laboratories may be directly compared regarding parasite density. The multiplex ddPCR was successful in quantifying the *Theileria* genus and *T. annulata* species. Field investigations from many countries have shown drug resistance to BPQ [6,8,9,39]. Our ddPCR-based technique may also be used to track instances of drug resistance by monitoring treatment response in BPQ-treated animals.

Since the findings of ddPCR are absolute and can be compared across labs without repetition, it is particularly effective in detecting bacteria, viruses, and parasites. ddPCR outperformed qPCR in identifying pathogen DNA in *E. coli*, *C. jejuni*, *CMV*, *C. trachomatis*, *Salmonella,* and *HIV* [23,24,25,26,27]. In the case of parasites such as *Plasmodium*, ddPCR was shown to be more sensitive and accurate than qPCR; however, in *Toxoplasma, Cryptosporidium, Leishmania*, *Trypanosoma*, *Theileria equi, Theileria cervi*, *Babesia, Bartonella*, and *Borrelia* species, ddPCR was found to be equivalent to qPCR [28,29,30,31,32,33,34,35].

## 5. Conclusions

In conclusion, we provided the first evaluation of the ddPCR platform for detecting the *Theileria* genus and *T. annulata* species in infected animals. The ddPCR test is recommended over qPCR because of its superior sensitivity and precision. Our test will be beneficial as a research instrument for detecting early or mixed infections, monitoring treatment response in the field, epidemiological studies, mass screening, and identifying carrier animals. Due to the inherent costs, ddPCR may not be economically feasible as a standard diagnostic technique at present. Nonetheless, ddPCR provides significant advantages, such as the ability to quantify DNA without calibration curves, which enables reliable and reference-free sample-specific results.

## Figures and Tables

**Table 1:** List of primers utilized for the study.

Primers	Sequence 5′-3′
Sense TaSP (F)	AAACCATTCGTGCCCAAG
Antisense TaSP (R)	CAGTCAAACGCTACAGTGA
Sense 18S rRNA (F)	ACAGGGAGGTAGTGACAAGA
Antisense 18S rRNA (R)	CGCTATTGGAGCCGGAATTA
**Probes**	**Sequence 5′-3′**
TaSP FAM	TCGCAATGTTGTGGATCATACTTCACA
18SRNA HEX	TATCAATTGGAGGGCAAGTCTGGTGC

## Data Availability

The datasets generated during and/or analyzed during the current study are available from the corresponding author on reasonable request.
